# Key Issues in the Modes of Action and Effects of Trichloroethylene Metabolites for Liver and Kidney Tumorigenesis

**DOI:** 10.1289/ehp.8692

**Published:** 2006-05-09

**Authors:** Jane C. Caldwell, Nagalakshmi Keshava

**Affiliations:** National Center for Environmental Assessment, Office of Research and Development, U.S. Environmental Protection Agency, Washington, DC, USA

**Keywords:** chloral hydrate, dichloroacetic acid, *S*-(1,2-dichlorovinyl)-l-cysteine, trichloroacetic acid, trichloroethanol, trichloroethylene

## Abstract

Trichloroethylene (TCE) exposure has been associated with increased risk of liver and kidney cancer in both laboratory animal and epidemiologic studies. The U.S. Environmental Protection Agency 2001 draft TCE risk assessment concluded that it is difficult to determine which TCE metabolites may be responsible for these effects, the key events involved in their modes of action (MOAs), and the relevance of these MOAs to humans. In this article, which is part of a mini-monograph on key issues in the health risk assessment of TCE, we present a review of recently published scientific literature examining the effects of TCE metabolites in the context of the preceding questions. Studies of the TCE metabolites dichloroacetic acid (DCA), trichloroacetic acid (TCA), and chloral hydrate suggest that both DCA and TCA are involved in TCE-induced liver tumorigenesis and that many DCA effects are consistent with conditions that increase the risk of liver cancer in humans. Studies of *S*-(1,2-dichlorovinyl)-l-cysteine have revealed a number of different possible cell signaling effects that may be related to kidney tumorigenesis at lower concentrations than those leading to cytotoxicity. Recent studies of trichloroethanol exploring an alternative hypothesis for kidney tumorigenesis have failed to establish the formation of formate as a key event for TCE-induced kidney tumors. Overall, although MOAs and key events for TCE-induced liver and kidney tumors have yet to be definitively established, these results support the likelihood that toxicity is due to multiple metabolites through several MOAs, none of which appear to be irrelevant to humans.

As discussed in the mini-monograph article on trichloroethylene (TCE) pharmacokinetics (Chiu et al. 2006b), exposure to TCE results in a complex internal mixture of parent compound and its metabolites [see Chiu et al. (2006b) for a postulated metabolism scheme]. Many of these metabolites have been shown to have toxicologic effects in TCE target organs, and several hypotheses have been put forth as to the “active” agent in TCE toxicity. Although TCE exposure has been associated with a wide array of adverse health effects, including neurotoxicity, immunotoxicity, developmental toxicity, endocrine toxicity, and several forms of cancer [see overview article by Chiu et al. (2006a)], most of the mechanistic information on TCE toxicity focuses on the liver and kidney. For liver toxicity and/or carcinogenesis, the TCE metabolites dichloroacetic acid (DCA) and trichloroacetic acid (TCA), both of which induce liver tumors in rodent laboratory studies, have been the major focus of study as potential active agent(s). For TCE-induced kidney effects, including cancer, glutathione (GSH) conjugation products such as *S*-(1,2-dichlorovinyl)-l-cysteine (DCVC) and its bioactivation products, some of which may be genotoxic and/or nephrotoxic (Lash et al. 2000), have been the major focus of study. An alternative hypothesis relating to the formation of formic acid has also been investigated (Dow and Green 2000).

The U.S. Environmental Protection Agency (EPA) 2001 draft TCE risk assessment (U.S. EPA 2001) concluded that it is difficult to determine which TCE metabolites may be responsible for liver and kidney tumorigenesis, the key events involved in their modes of action (MOAs), and the relevance of these MOAs to humans. In this article we present a review of the recent scientific literature on the MOAs and effects of TCE metabolites that may be related to TCE-induced liver and kidney tumorigenesis in the context of these key questions. Although some scientific conclusions can be drawn from this updated body of data, speculation as to the effect of these data on the final TCE risk assessment would be premature at this point because of the ongoing National Academy of Sciences consultation discussed in the overview article (Chiu et al. 2006a) and the subsequently planned revision of the EPA TCE risk assessment. Therefore, the purpose here and throughout this mini-monograph is to review recently published scientific literature in the context of how it informs the key scientific issues believed to be most critical in developing a revised risk assessment.

## TCE Metabolites and Liver Tumors

As discussed elsewhere in this mini-monograph (Chiu et al. 2006a; Scott and Chiu 2006), associations between TCE and liver tumors have been reported in both laboratory and epidemiologic studies. Three key issues with respect to liver tumor induction are the relative importance of TCA and DCA in tumor induction, the elucidation of key events in the hypothesized MOAs, and the relevance of TCE-, TCA-, and/or DCA-induced rodent liver tumors to human risk. Substantial new toxicologic data (although none on mutagenicity) derived from studies of TCA, DCA, and chloral hydrate (CH) inform all these issues.

### Dichloroacetic acid

As discussed in the pharmacokinetics article in this mini-monograph (Chiu et al. 2006b), direct evidence for the formation of DCA from TCE is limited because of the difficulty in directly detecting DCA after TCE exposure. However, Bull et al. (2002) have noted that DCA-induced liver tumors have been observed at exposure levels for which DCA cannot be detected *in vivo*. In addition, indirect evidence suggests that DCA is formed from TCE *in vivo* and contributes to liver tumor development. For instance, Schultz et al. (2002) and Bull et al. (2004) reported that pretreatment with either DCA or TCE inhibits the cytosolic enzyme glutathione *S*-transferase (GST) ζ , also known as maleylacetoacetate (MAA) isomerase, and TCA pretreatment does not. TCE treatment at 1 g/kg decreased the *V*_max_ for DCA metabolism to 49% of control levels, resembling effects of DCA treatment at 0.05 g/L (Schultz et al. 2002). The studies discussed in this section note a variety of DCA effects consistent with conditions that increase liver cancer risk across species and suggest pathologic changes induced by DCA on whole liver consistent with changes observed in preneoplastic foci from a variety of agents.

A number of studies suggest DCA-induced liver cancer may be linked to its effects on GST-ζ , which is part of the tyrosine catabolism pathway whose disruption in type 1 hereditary tyrosinemia has been linked to increased liver cancer risk in humans. Specifically, GST-ζ metabolizes MAA to fumarylacetoacetate (FAA), which displays apoptogenic, mutagenic, aneugenic, and mitogenic activities (Bergeron et al. 2003; Jorquera and Tanguay 2001; Kim et al. 2000), and FAA and MAA accumulation have been suggested to increase cancer risk (Tanguay et al. 1996). Cornett et al. (1999) reported DCA exposure in rats increased accumulation of maleylacetone (a spontaneous decarboxylation product of MAA), suggesting MAA accumulation. Ammini et al. (2003) reported depletion of GST-ζ to be exclusively a posttranscriptional event and suggested that genetic ablation of GST-ζ causes FAA and MAA accumulation in mice. Schultz et al. (2002) reported that DCA elimination is controlled by liver metabolism via GST-ζ in mice and that DCA inhibits its own metabolism, with young mice being the most sensitive. Conversely, older mice (60 weeks) overall had a decreased capacity to eliminate DCA compared with younger mice. The authors suggested that exogenous factors that deplete or reduce GST-ζ will decrease DCA elimination and may increase its carcinogenic potency. In humans, GST-ζ has also been reported to be inhibited by DCA and to be polymorphic (Blackburn et al. 2000, 2001; Tzeng et al. 2000). Board et al. (2001) reported one variant of GST-ζ to have significantly higher activity with DCA as a substrate than other isoforms, suggesting differential susceptibility.

Another area of investigation regarding the MOA for DCA liver tumor induction has been effects on glycogen and lipid metabolism. Individuals with glycogen storage disease or with poorly controlled diabetes have excessive storage of glycogen in their livers (glycogenosis) and increased risk of liver cancer (Adami et al. 1996; LaVecchia et al. 1994; Rake et al. 2002; Wideroff et al. 1997). In an animal model where hepatocytes are exposed to a local hyper-insulinemia, insulin induces formation of glycogenotic foci of altered hepatocytes (FAHs) in liver acini that develop into hepatocellular tumors (Evert et al. 2005). A number of studies have reported DCA-induced suppression of apoptosis, decreases in insulin, and glycogenosis in mouse liver at levels that also induce liver tumors (Bull 2004; Bull et al. 2004; Lingohr et al. 2001). Lingohr et al. (2002) reported that DCA-induced glycogenosis in isolated murine hepatocytes was dose related, occurred at very low doses (10 μM), occurred without the presence of insulin, was not affected by insulin addition, was dependent on phosphatidylinositol 3-kinase activity, and was not a result of decreased glycogen breakdown. The authors noted that phosphatidylinositol 3-kinase is also known to regulate cell proliferation and apoptosis in hepatocytes and that understanding these mechanisms may be important to understanding DCA-induced carcinogenesis. They also reported that insulin receptor (IR) protein levels decreased to 30% of controls in mouse liver after up to 52 weeks of DCA treatment. Activation of the IR is also the principal pathway by which insulin stimulates glycogen synthetase (the rate-limiting enzyme of glycogen biosynthesis). However, although DCA-induced liver tumors were glycogen poor, both IR protein and phosphorylation of mitogen-activated protein kinase (a downstream target protein of the IR) were elevated (Lingohr et al. 2001). The authors suggested that normal hepatocytes down-regulate insulin-signaling proteins in response to the accumulation of liver glycogen caused by DCA and that the initiated cell population, which does not accumulate glycogen and is promoted by DCA treatment, responds differently from normal hepatocytes to the insulin-like effects of DCA.

Glycogenosis and overexpression of proteins affected by the IR in preneoplastic foci are generalized responses that have been reported to be induced by a variety of agents. Nehrbass et al. (1998, 1999) reported that focal overexpression of IR substrate 1, a multisite docking protein occupying a central position in signaling cascades stimulated by a number of growth factors, including insulin, is an early event in hepatocarcinogenesis closely correlated with preneoplastic hepatic glycogenosis. IR substrate 1 is gradually down-regulated during progression of these lesions to a glycogen-poor neoplastic phenotype. Bannasch (2001) and Bannasch et al. (2001) describe the early phenotypes of preneoplastic foci induced by many oncogenic agents (DNA-reactive chemicals, radiation, viruses, transgenic oncogenes, and local hyperinsulinism) as insulinomimetic. Thus, the response of liver to DCA (glycogenosis with emergence of glycogen-poor tumors) is similar to the progression of preneoplastic foci to tumors induced from a variety of agents and conditions associated with increased cancer risk. Finally, Bull et al. (2002) reported that expression of IR was elevated in tumors of control mice or mice treated with TCE, TCA, and DCA but not in nontumor areas, which is consistent with this effect being non-specific to DCA.

Emerging studies of DCA-induced gene expression changes show alteration of a number of genes identified with cell growth, tissue remodeling, apoptosis, cancer progression, and xenobiotic metabolism in mouse liver at high drinking water doses (2 g/L DCA) (Thai et al. 2001, 2003). After 4 weeks, RNA expression was altered in four known genes [α_1_ protease inhibitor 3, cytochrome *b*_5_, stearoyl–CoA desaturase exon 6, and carboxylesterase] in two mice (Thai et al. 2001). Except for CoA desaturase, a similar pattern of gene change was reported in DCA-induced tumors (10 tumors from 10 different mice) after 93 weeks. Using cDNA microarray in the same mice, Thai et al. (2003) identified 24 genes with altered expression, 15 of which were confirmed by Northern blot analysis after 4 weeks of exposure. Of the 15 genes, 14 revealed expression suppressed 2- to 5-fold: *MHR 23A*, cytochrome P450 (CYP) 2C29, *CYP3A11*, serum paraoxonase/arylesterase 1, liver carboxylesterase, α_1_ anti-trypsin, *ER p72*, *GST*-π1, angiogenin, vitronectin precursor, cathepsin D, plasminogen precursor (contains angiostatin), prothrombin precursor, and integrin α 3 precursor. Additional genes, *CYP2A4*/5, had a 2-fold elevation in expression. After 93 weeks of treatment with 3.5 g/L DCA, Northern blot analyses of total RNA isolated from DCA-induced hepatocellular carcinomas showed similar alteration of expression (11 of 15). Interestingly, while genes involved in glycogen or lipid metabolism were not tested, the authors report no change in peroxisome proliferator–activated receptor α (PPARα ) and IR gene expression [see Keshava and Caldwell (2006) for additional discussion of PPARα ]. Although these studies do document genetic expression changes, a key event specific for DCA induction of tumors was not identified.

### Tumor phenotypes and the roles of TCA and DCA

Bull et al. (2002) reported that TCE-induced liver tumors in mice have phenotypic characteristics of both DCA- and TCA-induced tumors (i.e., a mixture of phenotypes with some tumors c-*jun* positive, some tumors c-*jun* negative, and some tumors displaying both). TCA-induced tumors did not stain with this antibody, whereas a number of DCA-induced tumors did. Moreover, mutation frequencies of the H-*ras* codon in TCE-induced tumors (1 g/kg/day) differed significantly from those induced by TCA (0.5 or 2 g/L), with DCA-induced tumors (0.5 or 2 g/L) intermediate between values obtained with TCA and TCE. The authors suggested that these data are not consistent with the hypothesis that all liver tumors induced by TCE are produced by TCA alone. However, use of the tumor phenotype to assign the MOA is less clear.

Although Bull et al. (2004) have suggested that the negative expression of c-*jun* in TCA-induced tumors may be consistent with a characteristic phenotype shown in general by peroxisome proliferators as a class, no evidence exists to support this. Tumors and foci induced by peroxisome proliferators have been suggested to have a phenotype of increased mitochondrial proliferation and mitochondrial enzymes (thyromimetic rather than insulinomimetic) (Keshava and Caldwell 2006). The studies cited in Bull et al. (2004) do not examine TCA-induced tumors for these properties. In addition, Bull et al. (2002) reported that TCA-induced tumors had a mutation frequency and spectrum of H-*ras* similar to those of historical controls, and the peroxisome proliferators ciprofibrate and methyl clofenapate caused lower *ras* mutation frequencies in mice. Miller et al. (1995) reported that “basophilic” preneoplastic lesions induced by the peroxisome proliferator Wyeth-14,643 (WY) alone or those initiated by *N*-nitrosodiethylamine and WY together do not express transforming growth factor(TGF)-α, whereas Bannasch (1996) reports increased TGF-α expression in preneoplastic lesions from agents other than peroxisome proliferators.

In addition, use of the term “basophilic” in describing preneoplastic foci or tumors can be misleading. The different types of FAHs have been related to three main preneoplastic hepatocellular lineages: the glycogenotic–basophilic cell lineage, its xenomorphic–tigroid cell variant, and the amphophilic–basophilic cell lineage. Specific changes of the cellular phenotype of the first two FAH lineages are similar in experimental and human hepatocarcinogenesis regardless of whether they are elicited by DNA-reactive chemicals, hormones, radiation, viruses, transgenic oncogenes, or local hyperinsulinism as described by the first two FAHs, and this similarity favors extrapolation of data obtained in animals to humans (Bannasch et al. 2001, 2003; Su and Bannasch 2003). In contrast, the amphophilic cell lineage of hepatocarcinogenesis has been observed mainly after rodents have been exposed to peroxisome proliferators or to Hepadnaviridae (Bannasch et al. 2001). However, a recent study of di(2-ethylhexyl) phthalate (DEHP) reported the majority of liver FAHs to be of the first two types (Voss et al. 2005) after a lifetime of exposure to DEHP with a dose-related tendency for increased numbers of amphophilic FAHs.

Bannasch (1996) described amphophilic FAHs and tumors induced by peroxisome proliferators to maintain the phenotype as the foci progress to tumors. They are glycogen poor from the start with increased numbers of mitochondria, peroxisomes, and ribosomes. The author further stated that other researchers’ “homogenous basophilic” descriptions of foci induced by WY are really amphophilic. Agents other than peroxisome proliferators can induce acidophilic or eosinophilic (due to increased smooth endoplasmic reticulum) or glycogenotic foci, which tend to progress to basophilic stages (due to increased ribosomes). Descriptions of tumors induced by TCA and DCA are consistent with this progression. Indeed, Pereira (1996) described the FAHs and tumors induced by DCA to be eosinophilic at higher exposure levels but, at lower or intermittent exposures, to be half eosinophilic and half basophilic. Regardless of exposure level, half the TCA-induced foci were eosinophilic, and half were basophilic, with tumors 75% basophilic. In control animals, the limited numbers of lesions were mostly basophilic; the rest were eosinophilic with the exception of a few mixed tumors.

Carter et al. (2003) examined the phenotype of liver tumors induced by DCA in mice and the shape of the dose–response curve for insight into its MOA. They reported a dose response of histopathologic changes (all classes of premalignant lesions and carcinomas) occurring in the livers of mice receiving from 0.05 to 3.5 g/L DCA for 26–100 weeks and suggested that foci and adenomas demonstrated neoplastic progression with time at lower doses than observed for DCA genotoxicity. Preneoplastic lesions were identified as eosinophilic, basophilic, and/or clear cell (grouped with clear cell and mixed cell) and dysplastic. Altered foci were 50% eosinophilic with about 30% basophilic. As foci became larger and evolved into carcinomas, they became increasingly basophilic. The pattern held true throughout the exposure range. An increase in atypical nuclei related to dose and length of exposure was also observed in “non-involved” liver. Glycogen deposition was also dose dependent with periportal accumulation in mice administered 0.5 g/L DCA. The findings are consistent with those of DeAngelo et al. (1999), who reported hepatocarcinogenicity at the lowest dose tested (0.05 g/L DCA or 8 mg/kg/day) and concluded that induction of liver cancer did not appear to be conditional on peroxisome proliferation (which was significantly increased only at much higher concentrations) or chemically sustained cell proliferation.

### DNA hypomethylation by TCA and DCA

Aberrant DNA methylation has emerged in recent years as a common hallmark of all types of cancers, with hypermethylation of the promoter region of specific tumor suppressor genes and DNA repair genes leading to their silencing (an effect similar to their mutation) and genomic hypomethylation (Ballestar and Esteller 2002; Berger and Daxenbichler 2002; Herman et al. 1998; Pereira et al. 2004; Rhee et al. 2002). Whether DNA methylation is a consequence or cause of cancer is a long-standing issue (Ballestar and Esteller 2002). Fraga et al. (2004, 2005) reported global loss of monoacetylation and trimethylation of histone H4 as a common hallmark of human tumor cells; they suggested, however, that genomewide loss of 5-methylcytosine (associated with the acquisition of a transformed phenotype) exists not as a static predefined value throughout the process of carcinogenesis but rather as a dynamic parameter (i.e., decreases are seen early and become more marked in later stages).

A body of work has also emerged regarding the hypothesis that toxicity of TCE and its metabolites may arise from its effects on DNA methylation status, although little is known as to the mechanism(s) by which this occurs. Tao et al. (2000) have reported that DCA (500 mg/kg), TCA (500 mg/kg), and TCE (1,000 mg/kg) decreased methylation of DNA and the promoter region of the protooncogenes c-*myc* and c-*jun* after 5 days of exposure in mouse liver. Increased levels of mRNA and protein for c-*myc* and c-*jun* were also reported after DCA and TCA treatment. Ge et al. (2001) reported DCA- and TCA-induced DNA hypomethylation and cell proliferation in the liver of female mice dosed at 500 mg/kg and decreased methylation of the c-*myc* promoter region in the liver, kidney, and urinary bladder. However, increased cell proliferation preceded hypomethylation. Hypomethylation of the c-*myc* gene in the liver has also been reported after exposure to the peroxisome proliferators 2,4-dichlorophen-oxyacetic acid (1,680 ppm), dibutyl phthalate (20,000 ppm), gemfibrozil (8,000 ppm), and WY (50–500 ppm, with no effect at 5 or 10 ppm) after 6 days in the diet (Ge et al. 2002). Although WY was much more potent as an inducer of cell proliferation and peroxisome proliferation than the other compounds tested, WY-induced hypomethylation was only slightly greater. Thus, the hypomethylation does not appear to be a chemical-specific effect at these concentrations.

After initiation by *N*-methyl-*N*-nitrosourea (25 mg/kg) and exposure to 20 mmol/L DCA or TCA (46 weeks), Tao et al. (2004) reported similar hypomethylation of total mouse liver DNA by DCA and TCA with tumor DNA showing greater hypomethylation. A similar effect was noted for differentially methylated region 2 of the insulin-like growth factor 2 (*IGF2*) gene The authors suggested that hypomethylation of total liver DNA and the *IGF2* gene found in nontumorous liver tissue would appear to be the result of a more prolonged activity and not cell proliferation, whereas hypomethylation of tumors could be an intrinsic property of the tumors. Overexpression of the *IGF2* gene in liver tumors and preneoplastic foci has been shown in both animal models of hepatocarcinogenesis and humans and may enhance tumor growth, acting via the overexpressed *IGF2* receptor (Scharf et al. 2001; Werner and Le Roith 2000). *IGF1* is the major mediator of growth hormone effects; it thus has a strong influence on cell proliferation and differentiation and is a potent inhibitor of apoptosis (Furstenberger and Senn 2002). Normally, expression of *IGF2* in the liver is greater during the fetal period than in the adult but is overexpressed in human hepatocarcinomas because of activation of fetal promoters (Scharf et al. 2001) and loss of imprinting (Khandwala et al. 2000). Takeda et al. (1996) reported that *IGF2* expression in the liver is monoallelic (maternally imprinted) in the fetal period, is relaxed during the post-natal period (resulting in biallelic expression), and is imbalanced in human hepatocarcinomas (leading to restoration of monoallelic *IGF2* expression).

However, Bull (2004) and Bull et al. (2004) have recently suggested that hypomethylation and peroxisome proliferation occur at higher exposure levels than those that induce liver tumors for TCE and its metabolites and that, therefore, these effects may not be key events in liver tumorigenesis. They reported that a direct comparison in the no-effect level or low-effect level for liver tumor induction in the mouse and several other end points shows that for TCA, liver tumors occur at lower concentrations than peroxisome proliferation *in vivo* but that PPARα activation occurs at a lower dose than either tumor formation or peroxisome proliferation. A similar comparison for DCA shows that liver tumor formation occurs at a much lower exposure level than peroxisome proliferation, PPARα activation, or hypomethylation. However, apoptosis is suppressed at levels of DCA exposure that also induce liver tumors as well as decreases in insulin and increases in glycogen. In addition, they reported that these chemicals are effective as carcinogens at doses that do not produce cytotoxicity.

### Chloral hydrate

A large fraction of TCE oxidative metabolism appears to go through CH, with subsequent metabolism to TCA and trichloroethanol (TCOH) (Chiu et al. 2006b). The recent studies of CH toxicity are consistent with the general presumption that oxidative metabolites are important for TCE-induced liver tumors, but whether CH and its metabolites are sufficient to explain all of TCE liver tumorigenesis remains unclear, particularly because of uncertainties regarding how DCA may be formed (Chiu et al. 2006b).

Recent studies of CH have been reported that may enable a comparison between toxicity of TCE and CH and may help elucidate its role in TCE effects. However, it is important to examine the strengths and weaknesses of such studies as well as their consistency and inconsistency with some of the proposed MOAs for TCE or its other metabolites. George et al. (2000) exposed male B6C3F_1_ mice and male F344/N rats to CH in drinking water for 2 years (up to 162.6 mg/kg/day). The authors considered all neoplastic lesions observed in the kidneys, spleen, and testes to be spontaneous for the male F344 rat and male B6C3F_1_ mouse based on historical controls, but data were not shown. In mice, preneoplastic foci and adenomas were increased in the livers of all CH treatment groups (13.5–146.6 mg/kg/day) at 104 weeks, the number of carcinomas was increased at the highest dose, and time to tumor decreased in all CH treatment groups. CH exposure did not alter serum chemistry, hepatocyte proliferation, or hepatic palmitoyl–CoA oxidase (an enzyme associated with PPARα agonism) in rats and mice at any of the time periods monitored. Study limitations included the following: only five animals were examined at the high dose, thereby limiting the study’s power to determine an effect; control mice had a high spontaneous carcinoma rate (54%), thereby limiting the ability to detect a treatment-related response; and no descriptions of the foci or tumor phenotype were given.

Leakey et al. (2003) studied the effects of CH exposure (0, 25, 50, and 100 mg/kg, 5 days/week, 104–105 weeks via gavage) in male B6C3F_1_ mice with dietary control used to manipulate body growth. Dietary control decreases background liver tumor rates (incidence of 15–20%) and is associated with decreased variation in liver-to-body weight ratios, thereby potentially increasing assay sensitivity. In dietary-controlled groups and groups fed *ad libitum*, liver adenomas and carcinomas (combined) were increased with overall tumor incidence reduced, and time to tumor increased after dietary control. In the dietary-restricted group administered 100 mg/kg CH, of the enzymes associated with PPARα agonism (total CYP, CYP2B isoform, CYP4A, or lauric acid ω-hydroxylase activity), only CYP4A and lauric acid ω-hydroxylase activity were increased at 15 months of exposure. No descriptions of liver pathology were given. Seng et al. (2003) described CH toxicokinetics in mice at doses up to 1,000 mg/kg/day for 2 weeks with dietary control and caloric restriction slightly reducing acute toxicity. Lauric acid ω-hydroxylase and palmitoyl–CoA fatty acid hydroxylase (another enzyme associated with PPARα agonism) were induced only at doses > 100 mg/kg in all groups, with dietary-restricted mice showing the greatest induction. Differences in serum levels of TCA, the major metabolite remaining 24 hr after dosing, did not correlate with hepatic lauric acid ω-hydroxylase activities across groups.

Ho et al. (2003) measured urinary isoprostan 8-epiprostaglandin F_2α_ , a marker of whole-body lipid peroxidation, and serum tumor necrosis factor (TNF)-α after administering injections of 100 mg/kg CH in corn oil to rats. A 5-fold elevation of 8-epiprostaglandin F_2α_ was observed on day 1, and serum TNF-α was elevated about 7-fold on day 2. Concurrent treatment with vitamin E (100 mg/kg) reduced the effects of CH treatment. In the same study, CH induced greater cytotoxicity but less apoptosis in human Chang liver cells than in human lymphocytes *in vitro*. The authors suggested a temporal relationship between lipid peroxidation and TNF-α induction in the rat and differential susceptibility between liver and extrahepatic tissue from these results.

## TCE Metabolites and Kidney Tumors

As was the case for liver tumors, associations between TCE and kidney tumors have been reported in both laboratory and epidemiologic studies (Chiu et al. 2006a; Scott and Chiu 2006). Studies of TCE-induced kidney tumorigenesis have concentrated mostly on metabolites formed in the GSH conjugation pathway of TCE metabolism. Under this set of hypotheses, DCVC, which is formed by γ-glutamyltransferase metabolism of the TCE GSH conjugate *S*-(1,2-dichlorovinyl)-l-glutathione, is thought to exert tumorigenic responses either directly or through bioactivation to reactive species in the kidney (Lash et al. 2003, 2005; Chiu et al. 2006b). Dow and Green (2000) have suggested an alternative mechanism of nephrotoxicity by which the oxidative TCE metabolites TCA and TCOH interact with vitamin B_12_ through a free radical mechanism to induce a B_12_ deficiency, leading to excessive formic acid. Substantial new toxicologic data derived from studies of DCVC and TCOH have emerged that may inform three key issues concerning the MOA for TCE-induced kidney tumors: an understanding of whether cytotoxicity plays a role in tumorigenesis, determination of the source(s) of cytotoxicity, and elucidation of species differences in susceptibility.

### S-*(1,2-Dichlorovinyl)-l-cysteine.*

DCVC has been used as a model compound in the study of proximal tubular (PT) cell injury (Nony and Schnellmann 2003; Vaidya et al. 2003a, 2003b, 2004) and also has been shown to induce effects on cell signaling at lower concentrations than cytotoxicity.

As a result of acute chemical insult, PT cells undergo mitochondrial dysfunction, ATP depletion, impaired solute and ion transport, loss of cell polarity, cytoskeletal disruption, and a variety of other effects. Injured PT cells that do not die or detach from the basement membrane are thought to contribute to regeneration of the tubular epithelium and to proliferate (Nony and Schnellmann 2003). Vaidya et al. (2003a, 2003b) reported PT necrosis after a single intraperitoneal (ip) injection of DCVC and mortality at doses of 40 mg/kg and greater; they also reported that prior exposure to DCVC (15 mg/kg, ip, for 72 hr) or mercuric chloride (6 mg/kg) was protective from DCVC-induced lethality after a second exposure (75 mg/kg, ip) in male mice. Vaidya et al. (2004) suggested that sustained stimulation of the renal extracellular-signal–related kinase pathway may have been a key factor in reported protection from acute DCVC toxicity. These studies show effects of DCVC *in vivo* but are of limited use in determining the potential MOA of kidney cancer induction reported in rats and epidemiology studies in humans (Scott and Chiu 2006) given the choice of a species insensitive to kidney tumor formation (mice) and the high doses used.

Using an *in vitro* model of primary cultures of PT and distal tubular (DT) rat kidney cells, Cummings et al. (2000) reported that unlike TCE, DCVC (0.1–1 mM) was highly cytotoxic in both PT and DT cells from short-term (3 hr) or long-term (72 hr) incubations, with DT cells having greater DCVC sensitivity after prolonged incubations. The authors suggested that PT cells are more susceptible to DCVC toxicity *in vivo* because they are first exposed to DCVC and have efficient transport into the cell. Chen et al. (2001) reported DCVC-induced apoptosis and mitochondrial effects in cultured porcine PT cells at similar concentrations (0.5 mM). However, Lash et al. (2001) reported that DCVC caused apoptosis and enhanced cell proliferation in primary cultures of human PT (hPT) cells at environmentally relevant doses and at earlier time points and lower concentrations than those that caused necrosis. The hPT cells from males were modestly more sensitive to DCVC-induced necrosis than were those from females (100 μM DCVC at 1 hr vs. 200 μM at 8 hr) with no sex-dependent change in apoptosis (10 μM at 2 hr). After a 24-hr treatment with as little as 10 μM DCVC, a small increase was also noted in the percentage of cells in S-phase, suggesting that cell proliferation was stimulated. The authors suggested that although rat PT cells may be more susceptible to DCVC-induced necrosis than humans, the opposite pattern is true for DCVC-induced apoptosis.

Lash et al. (2005) reported that DCVC altered hPT cell expression of proteins related to apoptosis (Apaf-1, α Bax, α PARP, Bcl-2, caspase-9), cell growth (c-*jun*, Hsp27, p53), and oxidative stress response (NF-κB p65 subunit), with patterns that are complex and dependent on concentration and exposure time. All types of proteins exhibited at least 200% increases at 10-μM concentration within 24 hr of incubation. Apoptosis was decreased at higher DCVC concentrations as necrosis was induced. Nony and Schnellmann (2001, 2003) reported 50% lethality in rabbit PT cells exposed to higher concentrations (200 μM DCVC), with surviving cells exhibiting irreversible inhibition of physiologic function. Addition of pharmacologic concentrations of l-ascorbic acid-2-phosphate that promote repair and proliferation, collagen IV that promotes repair of function but not proliferation, and other extracellular matrix proteins (fibronectin, laminin, and collagen I) had no effect. Nowak (2003) reports 25% rabbit PT cell death using the same model and decreased protein kinase C activity, mitochondrial function, and active Na^+^ transport. Van de Water et al. (2001) reported focal adhesion kinase in a porcine PT cell line (LLC-PK1) to be important in DCVC-induced apoptosis and concluded that loss of focal adhesion kinase activity results in perturbations of focal adhesion organization with subsequent inactivation of associated signaling molecules and loss of survival signal. Thus, several signaling pathways appear to be activated in response to DCVC that are dependent on time and exposure concentration as necrosis, apoptosis, or proliferation is induced.

DCVC can also be metabolized by the flavin-containing monooxygenase (FMO) system to produce DCVC sulfoxide (DCVCS), which is highly reactive and more nephrotoxic in rat PT cells than DCVC alone and plays a prominent role in effects on hPT cells (Lash et al. 2001, 2003). Lash et al. (2003) reported DCVCS-induced necrosis (200 μM at 48 hr) and apoptosis (within 1 hr at 10 μM exposure) in hPT cells, suggesting that DCVC bioactivation and cytotoxicity are more β-lyase dependent than FMO dependent at lower doses and early stages of exposure. Krause et al. (2003) reported polymorphism in FMO that may indicate a wide range of susceptibility to DCVCS in the human population.

### Trichloroethanol

Green et al. (2003, 2004) have investigated the hypothesis that formate formation from the TCE oxidative metabolite TCOH is a key event for TCE kidney effects; however, they have not provided much additional support because of data limitations and inconsistencies between reported effects of TCOH and those of TCE. Green et al. (2003) reported no dose response in formate levels in male Fischer rats after 4 weeks of exposure to TCOH in drinking water at 0, 0.5, and 1.0 g/L, so the lower dose was reduced to 0.35 g/L and folate was added to the drinking water for the rest of the 53-week exposure period. The authors suggested that these TCOH doses would result in formate levels of 0.5 g/L or greater (the upper range of TCE exposure levels in cancer bioassays). Using this paradigm, urinary formate levels were elevated at the two doses examined, peaking at 12 weeks of exposure, before declining, and were associated with decreased pH. Kidney-to-body weight ratios decreased throughout the time course of the study, with a smaller decrease in rats receiving TCOH at the highest level. Urinary *N*-acetyl glucosaminadase (NAG) levels were used as a marker of kidney damage along with creatinine, protein, alkaline phosphatase, and γ-glutamyl transferase. Only NAG was sporadically elevated throughout the study in both dose groups, with protein levels increasing at earlier time points. The authors reported some increase in cell replication in areas of the kidney at the highest dose at 29 weeks but not at 40 weeks. They also reported that kidneys showed treatment-related increases in α_2_μ-globulin (an effect not observed with TCE treatment) along with hyaline droplet accumulation and basophilia between 16 and 18 weeks but gave no quantitative analyses. By 40 weeks, tubular degeneration (increased eosinophilia, tubular vacuolation, and intratubular cast formation) was reported at the highest dose along with increased hyaline droplet accumulation and α _2_μ-globulin. By 52 weeks, these changes were no longer apparent, but foci of “atypical” tubule hyperplasia were reported in two rats in each TCOH-treated group. No tumors or preneoplastic foci were reported (unlike for TCE, which induces kidney tumors). The authors suggested that the pathologic changes reported with TCOH exposure were consistent with those exhibited by aged rats (chronic progressive nephropathy) and that increases in α_2_μ-globulin were not sufficient to account for hyaline droplet increases.

Green et al. (2004) examined kidney function in workers occupationally exposed to TCE and tried to relate it to TCOH toxicity. Estimates of TCE exposure levels were extrapolated from TCA levels in the urine that varied widely (range, 1–505 mg/L TCA). TCOH was also measured with a wide range of TCOH/TCA ratios between individuals (range, 0.6–20.1). In unexposed and TCE-exposed workers, a difference was observed in NAG, urinary albumin, and formate (2-fold increase over control levels) but not in other markers of urinary toxicity (α_1_μ- and β_2_μ-globulins, retinol binding protein, total protein, or GST-α ). A positive correlation was reported between TCA urine levels and GST-α but not NAG or albumin, in the TCE-exposed population. A correlation between formate and TCA and GST-α levels was reported. The authors did not present correlations between any parameters and urinary TCOH; thus, any inferences between TCOH concentration and toxicity are indirect. Interestingly, the mean concentration (as measured by milligrams per gram creatinine) of urinary TCA (72 ± 84 mg/g) was higher than that of formate (9.45 ± 4.78 mg/g) in the TCE-exposed group.

## Summary

The studies reviewed here on effects of TCE metabolites provide important insights into TCE-induced liver and kidney tumorigenesis. A number of the studies suggest that both DCA and TCA contribute to TCE-induced liver tumors and that many DCA effects are consistent with conditions that increase liver cancer risk and with changes in neoplasia. Studies of DCVC and its bioactivation products have revealed a number of different possible cell signaling effects that may be related to kidney tumorigenesis at lower concentrations than those that produce cytotoxicity in the kidney. Although MOAs and key events for TCE-induced liver and kidney tumors have yet to be definitely established, most of the studies described here suggest that multiple metabolites may contribute to liver and kidney tumorigenesis through a number of MOAs, none of which appear to be irrelevant to humans.
